# Prognostic value of applanation tonometry, pachymetry and automated perimetry in monitoring glaucoma progression: A longitudinal analytical study

**DOI:** 10.6026/973206300222244

**Published:** 2026-04-30

**Authors:** Shivam Pandey, Kritika Upadhyay, Shubhi Jain, Shreyashi Tripathi, Sachin Parmar

**Affiliations:** 1 Department of Ophthalmology, NSCB Medical College, Jabalpur, Madhya Pradesh, India; 2 Department of Community Medicine, V.K.S. Government Medical College, Neemuch, Madhya Pradesh, India

**Keywords:** Glaucoma, intraocular pressure (IOP), central corneal thickness (CCT), automated perimetry, visual field loss

## Abstract

Glaucoma is a leading cause of irreversible blindness and accurate monitoring using intraocular pressure (IOP), central corneal thickness (CCT) and visual field analysis is essential for early detection of progression. This 24-month longitudinal analytical study evaluated the prognostic value of Goldmann applanation tonometry, pachymetry and automated perimetry in patients with primary open-angle, primary angle- closure and secondary glaucomas. Mean IOP was high in PACG and secondary angle-closure glaucoma, while CCT was greatest in secondary open- angle glaucoma and PACG and visual field loss was severe in POAG. Adjusting IOP for CCT yielded consistently lower corrected values, emphasizing the importance of pachymetry in refining glaucoma assessment and prognosis. Thus, data support a personalized, multimodal approach to glaucoma monitoring integrating IOP, CCT and visual field parameters to optimize long-term visual outcomes.

## Background:

Glaucoma is a chronic progressive optic neuropathy and a leading cause of irreversible blindness worldwide, with functional loss often remaining asymptomatic until advanced stages [1]. In this context, accurate monitoring of disease progression is essential to prevent disability and preserve quality of life over long-term follow up [2]. Intraocular pressure (IOP) remains the only modifiable risk factor with robust evidence for slowing glaucomatous damage and treatment strategies still largely revolve around achieving and maintaining individualized target IOP levels [3]. Failure to remain at or below clinician-set target IOP has been associated with faster rates of retinal nerve fiber layer (RNFL) thinning, underscoring the prognostic significance of longitudinal IOP control [4]. Goldmann applanation tonometry (GAT) is considered the clinical gold standard for IOP measurement but assumes an average corneal thickness and biomechanical profile, which may not hold true in many glaucoma patients [5]. Variations in central corneal thickness (CCT) and corneal biomechanical properties can introduce clinically relevant errors in applanation readings, potentially leading to under or overestimation of true IOP and misclassification of progression risk [6]. Central corneal thickness has itself emerged as an important parameter in glaucoma assessment, with thinner corneas consistently associated with a higher likelihood of glaucomatous damage and faster visual field loss in susceptible eyes. Recent work suggests that CCT may act as a surrogate for the biomechanical susceptibility of posterior ocular tissues, providing additional prognostic information beyond IOP alone [2]. Long-term cohort data in patients with advanced open-angle glaucoma have identified lower CCT and inadequate IOP reduction as significant risk factors for structural and functional progression, supporting the combined prognostic value of pachymetry and tonometric measurements over extended follow up. Such findings highlight the need to integrate CCT-adjusted IOP interpretation into routine monitoring algorithms in moderate to advanced disease [7]. Standard automated perimetry (SAP) remains the mainstay functional test for detecting and monitoring visual field (VF) damage in glaucoma, forming the basis of most clinical decisions on treatment escalation or surgical intervention.

Optimization of SAP testing strategies, including stimulus parameters and test frequency, is a major focus of current research aimed at improving sensitivity to early and subtle progression [8]. Visual field progression has been strongly linked to subsequent deterioration in glaucoma-related quality of life, with both baseline damage and early rates of VF change predicting long-term disability [9]. Estimating visual field rates of change using longitudinal SAP data is therefore widely adopted as a practical approach to stratify patients by risk and to identify those with rapid progression who may require more aggressive therapy. Contemporary longitudinal studies have reinforced that timely detection of disease progression, using both structural and functional metrics, remains an unmet need in glaucoma management [10]. Emerging analytic approaches leveraging serial testing emphasize that early recognition of faster VF or RNFL deterioration provides a critical window for intervention before severe visual impairment occurs [11]. Front-loaded visual field testing protocols, which employ more frequent SAP examinations early in follow up, have demonstrated higher detection rates of glaucomatous progression and shorter time to identifying significant mean deviation slopes compared with conventional annual testing schedules. These results support the concept that optimizing the timing and intensity of automated perimetry can enhance prognostic assessment and guide individualized monitoring intervals [18]. Despite the availability of structural imaging, visual field testing remains the reference standard for functional evaluation and guided progression analysis (GPA) tools are widely used to flag likely progression events based on serial SAP data [13]. The research fills the knowledge gap by proving that no individual parameter is sufficient but the interrelation of IOP (corrected by CCT) and visual field measures, stratified by glaucoma subtype is the most clinically significant basis of monitoring and long-term optimization of visual outcome. Therefore, it is of interest to evaluate the prognostic value of applanation tonometry, pachymetry and automated perimetry in monitoring glaucoma progression over time.

## Methodology:

## Study design:

This study is a hospital-based, longitudinal analytical research conducted over a period of 24 months at a tertiary eye care center. The study adhered to the ethical guidelines set by the Declaration of Helsinki and was approved by the Institutional Ethics Committee. Informed consent was obtained from all participants prior to inclusion in the study.

## Study population:

The study included adult patients (18 years and older) diagnosed with glaucoma, including primary open-angle glaucoma (POAG), primary angle-closure glaucoma (PACG) and secondary glaucoma. The inclusion criteria consisted of patients with confirmed glaucoma who had a history of adequate follow-up and were willing to participate in repeated assessments over the study duration. Patients with ocular media opacities, conditions causing unreliable intraocular pressure (IOP) measurements (*e.g.*, corneal scarring) and those who had undergone previous glaucoma surgery were excluded.

## Inclusion criteria:

Adults aged 18 years and older, diagnosed with POAG, PACG or secondary glaucoma, confirmed by clinical and instrumental examinations, at least one year of clinical follow-up before enrollment, ability and willingness to comply with the study protocol, including repeated visits for IOP, pachymetry and perimetry assessments.

## Exclusion criteria:

Ocular media opacities preventing accurate tonometry or perimetry (*e.g.*, dense cataracts), previous ocular surgery unrelated to glaucoma (*e.g.*, refractive surgery), pregnancy or systemic conditions affecting vision (*e.g.*, advanced systemic diseases), inability to attend follow-up visits due to mobility issues or noncompliance.

## Data collection:

The study involved a detailed clinical assessment at baseline and subsequent follow-up visits every 6 months over a 24-month period. The following data were collected:

## Demographic and clinical data:

[1] Age, gender and ethnicity

[2] Medical history, including systemic diseases (*e.g.*, diabetes, hypertension)

[3] Family history of glaucoma

## Intraocular pressure (IOP) measurement:

IOP was measured using Goldmann applanation tonometry at each visit. Three measurements were taken and the average value was recorded for analysis. Tonometry was performed under standard conditions, with the patient in a seated position and anesthesia applied to the cornea.

## Pachymetry (Central Corneal Thickness - CCT):

Pachymetry was performed using an ultrasound pachymeter to measure the central corneal thickness (CCT) in micrometers. CCT was measured at baseline and then annually for the duration of the study. The relationship between CCT and glaucoma progression was analyzed as a potential risk factor for optic nerve damage.

## Automated perimetry (visual field testing):

[1] Automated perimetry was performed using the Humphrey Field Analyzer (HFA), 24-2 SITA Standard test protocol. Visual field testing was conducted at baseline, 12 months and 24 months. The pattern of visual field defects, such as paracentral scotomas, arcuate defects and overall field loss, was recorded.

[2] Perimetric progression was defined by a worsening of the mean deviation (MD) or pattern standard deviation (PSD) compared to baseline values, with a focus on changes over time.

## Follow-up:

Participants were seen at 6-month intervals for a total of four follow-up visits over the 24-month study period. During each visit, the following procedures were repeated:

IOP measurement: Using Goldmann applanation tonometry

Pachymetry: To assess CCT

Automated perimetry: To track changes in visual field

## Outcome measures:

The primary outcome measure was the rate of glaucoma progression, as determined by the following:

[1] IOP changes: Fluctuations in intraocular pressure and their correlation with disease progression.

[2] CCT changes: The role of central corneal thickness in determining the risk of glaucoma progression, especially in relation to IOP.

[3] Visual field changes: The progression of visual field defects, including the development of new defects or worsening of existing ones. Progression was defined based on changes in MD and PSD values, along with qualitative visual field defect analysis.

## Ethical considerations:

The study adhered to the ethical standards outlined in the Declaration of Helsinki. Ethical approval was obtained from the Institutional Ethics Committee and all participants provided written informed consent before enrollment. Participants were assured of their right to withdraw from the study at any time without consequences to their care.

## Results and Discussion:

The results of the intraocular pressure (IOP), central corneal thickness (CCT) and visual field parameters analysis in all the glaucoma subtypes showed significant intergroup differences, which is a complete picture of the prognostic role of these parameters in the monitoring of glaucoma progression and the necessity of managing all the subtypes in different ways. The mean IOP of the primary open-angle glaucoma (POAG) patients was less than the primary angle-closure glaucoma (PACG) and secondary angle-closure glaucoma (Secondary ACG) groups, where the mean IOP of patients was 23.94 ± 3.84 mmHg and 26.40 ± 3.41 mmHg and 26.41 ± 1.31 mmHg, respectively (Table 1 - see PDF). IOP fluctuation was also a major observation with PACG and Secondary ACG having considerably higher mean IOP values than POAG and Secondary OAG. This finding is in line with Liu *et al.* [14] who proved that glaucoma types of angle-closure have high IOP as a result of anatomical constrictions or blockage of the anterior chamber angle to cause pressure spikes. Increased IOP and its changes have been continuously attributed as a key risk factor affecting the development of glaucoma, especially in PACG and POAG patients and the need to regulate changes in IOP variability to reduce the effect of optic nerve damage. The assessment of central corneal thickness showed that the mean cornea of Secondary open-angle glaucoma (Secondary OAG) patients (569.96 ± 11.37 µm) and PACG patients (567.47 ± 11.01 µm) had the highest and lowest centrally located corneal thickness respectively. Secondary ACG patients (556.70 ± 19.89 µm) had the lowest centrally located corneal thickness (Table 2 - see PDF). CCT highly affected the measurements of IOP among patients with glaucoma, whereby corneas were found to be thicker in patients with Secondary OAG and PACG compared to POAG and Secondary ACG. This is in support of research findings by Kohlhaas *et al.* [15] and Patwardhan *et al.* [16] that inadequate attention to CCT could result in underestimation or overestimation of actual IOP values and thus give misguided clinical judgment. Rojas *et al.* [17] found that reduced CCT was also confirmed as an independent marker of advanced functional loss. The corrected IOP values after the adjustment of corneal thickness were marginally lower than the measured IOPs in all groups and PACG and Secondary ACG had higher mean corrected IOPs than POAG or Secondary OAG (Table 3 - see PDF), indicating the indispensable importance of pachymetry in the correction of IOP values Kwon *et al.* [18] further reported that eyes with higher corrected IOP demonstrated increased rates of progression compared to uncorrected measurements, highlighting the necessity of adjusting for corneal thickness when interpreting tonometric data for prognosis and management decisions. This nuanced approach helps avoid under-treatment or over-treatment based on potentially misleading raw IOP values and clinicians are urged to incorporate CCT measurements routinely to optimize diagnosis and monitor progression accurately. Mean deviation (MD) values on automated perimetry showed the greatest visual field loss in POAG (-8.24 ± 4.75 dB), followed by PACG (-7.88 ± 4.90 dB), whereas Secondary ACG patients had the mildest loss (-3.01 ± 1.26 dB) (Table 4 - see PDF). Assessment of visual field loss severity further confirmed that most POAG patients had moderate or severe defects, whereas all Secondary ACG patients had only mild impairment (Table 5 - see PDF). The visual field defects were more chronic and pronounced in POAG patients and this is in line with its insidious and chronic nature that makes the patients realize the defects late enough when a significant amount of damage has been caused. PACG patients too suffered large visual field losses but not as serious and it is consistent with the previous study, which indicates the possibility of the intervention after the appearance of acute forms to ameliorate the effects of the long-term field losses. Secondary ACG patients on the other hand reported the least field loss, which may be due to early diagnosis and treatment of the disease under the etiological condition of acute or secondary and thus, POAG and PACG showed more progressive visual field loss than secondary glaucomas. These variations are associated with different pathophysiological processes and natural courses that require specific monitoring regimes and unique management plans. Individualized therapy that considers the differences in IOP profiles, CCT and functional field loss has the best probability of achieving better clinical outcomes with all types of glaucoma.

## Conclusion:

We show the need for integrating applanation tonometry, pachymetry and automated perimetry into routine glaucoma management for effective longitudinal monitoring. Accurately measured and adjusted IOP combined with careful functional assessment enables early detection of progression, guiding timely intervention to preserve vision. However, future larger-scale, long-term studies are warranted to validate these findings and refine guidelines for personalized glaucoma care.
